# Conversation dynamics in a multiplayer video game with knowledge asymmetry

**DOI:** 10.3389/fpsyg.2022.1039431

**Published:** 2022-11-03

**Authors:** James Simpson, Patrick Nalepka, Rachel W. Kallen, Mark Dras, Erik D. Reichle, Simon G. Hosking, Christopher Best, Deborah Richards, Michael J. Richardson

**Affiliations:** ^1^School of Psychological Sciences, Macquarie University, Sydney, NSW, Australia; ^2^Centre for Elite Performance, Expertise and Training, Macquarie University, Sydney, NSW, Australia; ^3^School of Computing, Macquarie University, Sydney, NSW, Australia; ^4^Human and Decision Sciences Division, Defence Science and Technology Group, Melbourne, VIC, Australia

**Keywords:** team coordination, multiplayer video game, conversation, recurrence quantification analysis, team situation awareness, role asymmetries

## Abstract

Despite the challenges associated with virtually mediated communication, remote collaboration is a defining characteristic of online multiplayer gaming communities. Inspired by the teamwork exhibited by players in first-person shooter games, this study investigated the verbal and behavioral coordination of four-player teams playing a cooperative online video game. The game, *Desert Herding*, involved teams consisting of three *ground players* and one *drone operator* tasked to locate, corral, and contain evasive robot agents scattered across a large desert environment. Ground players could move throughout the environment, while the drone operator’s role was akin to that of a “spectator” with a bird’s-eye view, with access to veridical information of the locations of teammates and the to-be-corralled agents. Categorical recurrence quantification analysis (catRQA) was used to measure the communication dynamics of teams as they completed the task. Demands on coordination were manipulated by varying the ground players’ ability to observe the environment with the use of game “fog.” Results show that catRQA was sensitive to changes to task visibility, with reductions in task visibility reorganizing how participants conversed during the game to maintain team situation awareness. The results are discussed in the context of future work that can address how team coordination can be augmented with the inclusion of artificial agents, as synthetic teammates.

## Introduction

Conversing with others involves a convergence of language (Clark, 1996) as well as a synchronization of bodies (Hoehl et al., 2021) which enables a shared understanding of a topic or a task at hand. Recent research has documented how the coordination of minds and bodies during conversation can be disrupted during remote online interactions (Tomprou et al., 2021). Although the transition to remote work during, for example, the COVID-19 pandemic has been disruptive for many people (Yang et al., 2021), remote collaboration is a defining characteristic of online multiplayer game communities. In multiplayer first-person shooters (FPS) specifically, the voice channel is the primary means of communication between team members as the expressivity of non-verbal communication, such as gesturing, is limited (Tang et al., 2012). Additionally, like teams in the workplace, many multiplayer video games consist of players taking on specialized roles that uniquely contribute to a team’s success (e.g., “tanks” and “healers” in games such as *World of Warcraft* and *Overwatch*). Further, these teams can either be laterally (e.g., *Overwatch*) or hierarchically organized (e.g., military simulation games like *ARMA 3* or *Squad*). Thus, online multiplayer video games provide a rich platform to investigate the processes which enable effective team performance, coordination, and communication.

Within the psychological sciences, video games have become a popular platform to study cognition due to their behavioral richness and ability to collect large participant samples (Stafford and Dewar, 2014; Griffiths, 2015; Gray, 2017; Brändle et al., 2021). However, most of this work has focused on individual cognition in solitary games, and less on the interactive processes which enable remote group collaboration to be possible. A notable exception is the work of Woolley, Malone and colleagues (Woolley et al., 2010; Riedl et al., 2021) which has explored the “collective intelligence” factor framework – a team analogue to the concept of general intelligence – to understand the attributes differentiating high versus low performing teams using the popular video game *League of Legends* (Kim et al., 2017). A major determinant of the collective intelligence of teams is the average social perceptiveness of team members (Woolley et al., 2010; Riedl et al., 2021), highlighting the importance of social responsivity and interactivity within teams. Another recent example is the work by Guastello and colleagues (Guastello et al., 2022) who used *Counter-Strike* to investigate the relationship between team cohesion and autonomic synchrony on team performance when facing against a team of artificial agents (or “bots”).

The capacity for teams to interact and coordinate effectively together is hypothesized to relate to the team possessing “team situation awareness” (TSA; Gorman et al., 2006). The term “situation awareness” (SA), historically, has been defined as “the perception of elements in the environment within a volume of time and space, the comprehension of their meaning, and the projection of their status in the near future” (Endsley, 1988, p. 97; see also Gorman et al., 2006). Within a team context, TSA refers to each team member possessing “the situation awareness required for his/her job” (Endsley, 1995; see also Demir et al., 2017; McNeese et al., 2021).

From an “interactive team cognition” (ITC) framework (Cooke et al., 2013), teams possess TSA following the formation of “interpersonal synergies” (Riley et al., 2011) or coordination strategies that are robust to perturbations. Interpersonal synergies are lawful couplings between individuals that adapt to variation in task context due to naturally occurring motor variability (Romero et al., 2015) or differences in constraints due to task (Ramenzoni et al., 2011; Abney et al., 2015; Davis et al., 2017) or physical (Schwab et al., 2021) demands. The ITC framework is consistent with understanding teams as complex adaptive systems (McGrath et al., 2000; Ramos-Villagrasa et al., 2018).

**Figure 1:**
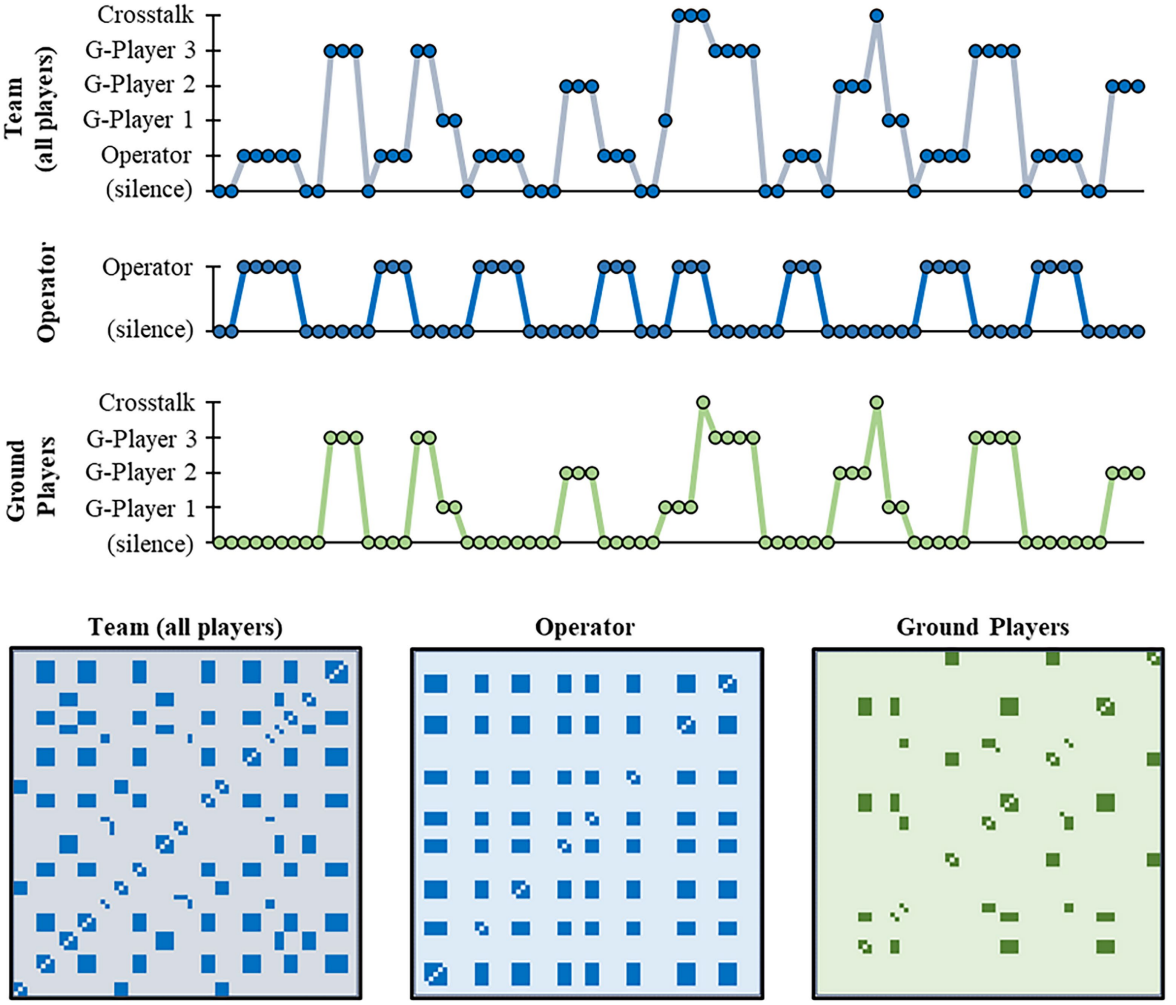
Illustration of categorical recurrence quantification analysis (catRQA). (top) Illustration of the different player communication data series analyzed using catRQA. (bottom) Illustration of the corresponding recurrence plot (RP) for each data series type. See text for more details.

Of particular relevance here is that the social coordination between team members and TSA can be assessed using various recurrence-based methods (Marwan et al., 2007; Richardson et al., 2014; Gorman et al., 2020). Specifically, recurrence quantification analysis (RQA) is an analysis technique that describes the coupling dynamics between two systems (e.g., two people having a conversation), or a description of a single system’s behavior (e.g., a team) such as whether the behaviors are stable or deterministic. These descriptions are derived by computing statistics from generated recurrence plots (RPs) of the inputted behavioral or event time series (e.g., Figure 1 in Method). RQA has been applied to understand, for example, the interaction dynamics of interpersonal postural coordination during conversation (Shockley et al., 2003, 2009), gaze behavior (Richardson and Dale, 2005), coordination during musical performance (Proksch et al., 2022) and human-machine interaction (Demir et al., 2019; Nalepka et al., 2021a). RQA has also been applied to task contexts where participants are homogenous in their roles, or where specific roles are assigned (Louwerse et al., 2012), or where there are differences between participants in ability (Davis et al., 2017; Schwab et al., 2021). Although typically unidimensional time series are used to construct RPs, recent work has extended RQA to the analysis of multidimensional time series data (Wallot et al., 2016). Within the social coordination and TSA literature, RQA has been applied to team problem-solving in tasks such as aerial reconnaissance (Demir et al., 2019) and intensive care units and submarine crews (Gorman et al., 2020). The results of this research have demonstrated that measures derived from RQA (in particular, %DET, which measures how often behavioral sequences are repeated [see Method]), is sensitive to a team’s skill level, or changes to task context that negatively impacts TSA (e.g., an unexpected event).

In competitive multiplayer games, verbal communication amongst teammates is constant and is used to establish and maintain TSA (Tang et al., 2012). These verbal statements (i.e., “callouts”) consist of providing up-to-date information about the locations of opposing players and are oftentimes directed to the entire team – as opposed to specific players. In competitive games which allow eliminated players to observe their teammates’ point of view (i.e., “spectate”), eliminated players do not sit idly but instead contribute a greater share to making callout statements regarding the locations of opponents. The role of the spectator is like the role of a drone operator in providing critical information to military personnel.

Members taking on specific roles can influence a team’s overall functioning and performance (e.g., “brokers” in social networks; Jackson, 2019). Individual roles are capable of scaffolding interactions in the case of caregiver-infant interactions (e.g., Fusaroli et al., 2021), compensate for task-related burdens placed on certain individuals (Davis et al., 2017), or dictate the temporal order of turn-taking (Abney et al., 2015). Understanding how roles impact team functioning and the development of TSA is also important if, for example, the goal is to design artificial agents that can be embedded within teams to perform specific roles to enhance team collaboration.

The current study explored how specific roles contribute to team coordination and communication in a custom-made multiplayer video game which consisted of game mechanics familiar to commercial FPS games. In the task employed here, teams of three individuals had to coordinate to search for and corral evasive agents which were scattered across a large virtual environment. Participants had a “first-person” perspective of the game and utilized keyboard and mouse controls common in the FPS genre (Nalepka et al., 2022). Inspired by the role of the “spectator” in multiplayer FPS games, a fourth player was introduced who served as a spectator or *drone operator* in the task. This individual was able to observe the views of the three participants (referred to as *ground players*), as well as have access to a map that provided veridical information about the ground players’ positions, locations of the agents that needed to be corralled, and the location where the agents needed to be corralled into.

Of particular interest was the communication dynamics that emerge between the drone operator and ground players when completing the task, and whether these dynamics relate to team performance. The necessity for the operator was manipulated by varying the ability of ground players to directly perceive their environment when completing the task. This was done with the use of environmental “fog” which obscured the ground players’ vision, but not the operator’s ability to provide task-relevant information with the use of the game map. This experiment was part of a larger study which included two additional task manipulations. First, the number of agents that needed to be corralled was manipulated (9 or 18). Second, on certain trials, an additional agent would randomly appear towards the end of the trial. The number of targets was not expected to impact the structure of the conversation dynamics (but may impact the quantity of statements), and the appearance of new agents to corral was expected to elicit greater communication from the drone operator to notify the ground players.

Categorical RQA (catRQA), a discretized version of RQA, was utilized to investigate the communication dynamics of the team, as well as the interaction dynamics between the ground players and the drone operator separately. The expectation was that an increase in task difficulty (e.g., the presence of fog) would result in a greater magnitude of conversation between team members, as well as more structured patterns of communication (as assessed by %DET from catRQA). This increase in communication and communication structure was expected to reflect the greater necessity to maintain effective TSA to ensure task success, and in particular, between ground players and the drone operator, who was not affected by the task difficulty manipulations and can therefore provide accurate information about the states of the task environment.

As alluded to above, this experiment is part of a much larger study. The overall aim of the larger study is to model the actions and communicative behaviors of teams engaged in complex and dynamically evolving task contexts for the development of human-inspired artificial agents which can be embedded within such teams. A potential application of this work is to reduce the demands associated with team training exercises by reducing the number of personnel needed (Rigoli et al., 2022). In order to achieve this aim, we first must understand the contextual factors, behavioural strategies and communication dynamics which impact team performance and explains expertise. This experiment is one of the first of many which seeks to answer this question (Nalepka et al., 2022).

## Materials and methods

### Recruitment criteria

Recruitment materials were distributed *via* e-mail to senior undergraduate Psychology, Cognitive Science and Computer Science students at Macquarie University, and *via* social media posts to groups targeting undergraduate students. Interested individuals were asked to complete a survey where demographic information (e.g., age, gender), availability (e.g., days/times during the week), computer hardware and software specifications, and internet speed information were collected. Potential participants who completed the interest survey were contacted if their personal computer met the following minimum requirements: Windows 10 or MacOS Mojave 10.13, with at least 8 GB of system memory (RAM), an Intel Core i5 or equivalent CPU, a minimum display resolution of 1,280 × 720 (HD), and a responsive internet connection (i.e., a ping rate under 100 ms, tested using).^1^

### Participants

Forty individuals (*M* age = 23.73 years, 35 right-handed) were selected to participate in the study and received gift vouchers for their participation. All participants were either native (*N* = 29) or fluent (*N* = 11) English speakers. Twenty-nine (72.5%) of the participants reported to play video games weekly (for any duration), with 10 (25%) participants reporting that they play video games for at least 20 h per week. The participants were grouped into ten, four-person teams. Team assignment was determined by matching participant day and time availability, and all teams included at least one female participant (the sample consisted of 20 female, 19 male and one non-binary participant). The resultant teams had the follow gender composition: two teams with one female/three male, five teams with two female/two male, two teams with three female/one male, and one team consisting of two female participants, a male participant, and a non-binary participant.

The study was conducted entirely online due to restrictions imposed by the COVID-19 pandemic at the time of data collection, with participants completing the experiment using their own Windows or MacOS computer. All participants had to complete the study from Australia (all participants were either in NSW or Victoria) to ensure minimal internet delay (*M* ping rate = 10.4 ms). To facilitate verbal communication, each participant was required to have a microphone available. Participants were also asked to use a computer mouse (as opposed to, for example, a trackpad) to enable greater control of their in-game avatars.

### Materials and design

#### Desert herding game

Teams played a networked multiplayer game referred to as *Desert Herding* (Nalepka et al., 2022). The task was inspired by previous research exploring corralling behaviors in two-person task contexts (Nalepka et al., 2017, 2019, 2021b; Rigoli et al., 2020). The game was designed using the Unity3D Game Engine (Version 2018.4 LTS; Unity Technologies, San Francisco, CA, United States). The game server was hosted on Amazon Web Services (AWS) EC2 with Windows 10 (server located in Sydney). Participants downloaded a standalone version of the game to their personal/home computer and connected to the server as clients. Game states, including participant movements, were transmitted across the network using Mirror (vis2k)^2^ networking architecture. The game could only be played when the experimenter instantiated a game session on the server, and thus participants could only play the game and complete the experiment during their allocated sessions. The server recorded all game state data at a sample rate of 90 Hz. In addition to interacting with each other in-game, participants could verbally communicate *via* the use of internet teleconferencing software (Zoom Video Communications Inc., San Jose, California; set to audio-only communication).

**Figure 2:**
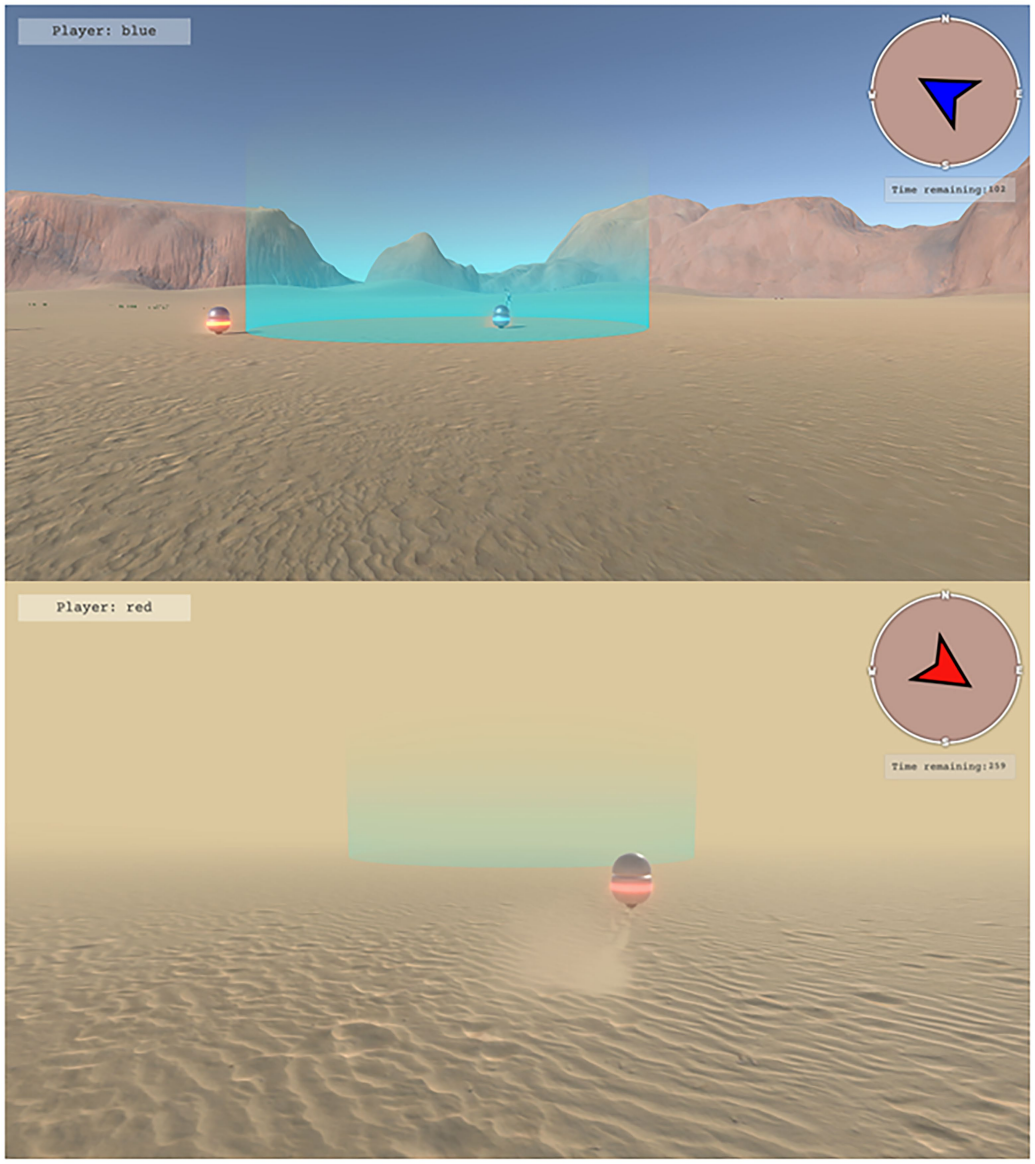
Ground player view of task manipulations. Participants playing as ground players used keyboard and mouse controls to navigate their humanoid avatars across a large desert environment. Teams consisted of three ground players who were tasked to search, corral, and contain target agents (TAs) within the cyan containment area. Ground players’ ability to observe the environment was manipulated by controlling the absence (top) or presence (bottom) of environmental fog. Participants either had full visibility of the desert environment (top), or their vision was obscured by fog (bottom). Ground players had access to a compass (top right of each image), a timer indicating how much time was remaining in a trial, and a nametag (top left of each image) reminding the participant of their name in the experiment.

The game involved locating, corralling, and containing a set of robots or target agents (TAs; the spherical robots in Figure 2) who freely roamed about a large area of desert terrain (500 × 500 m in game space). The goal of the game was for teams to work together to locate, corral, and contain the TAs within a fixed central containment area (cyan highlighted circle in Figure 2, measuring 10 m in diameter). Participants were assigned to one of two distinct roles. Three participants completed the task as *ground players*, while the fourth participant played the role of *drone operator*. Game avatars were identifiable by a unique color assigned to each participant (red, blue, black, white). The color was also the name participants used in the experiment.

**Figure 3:**
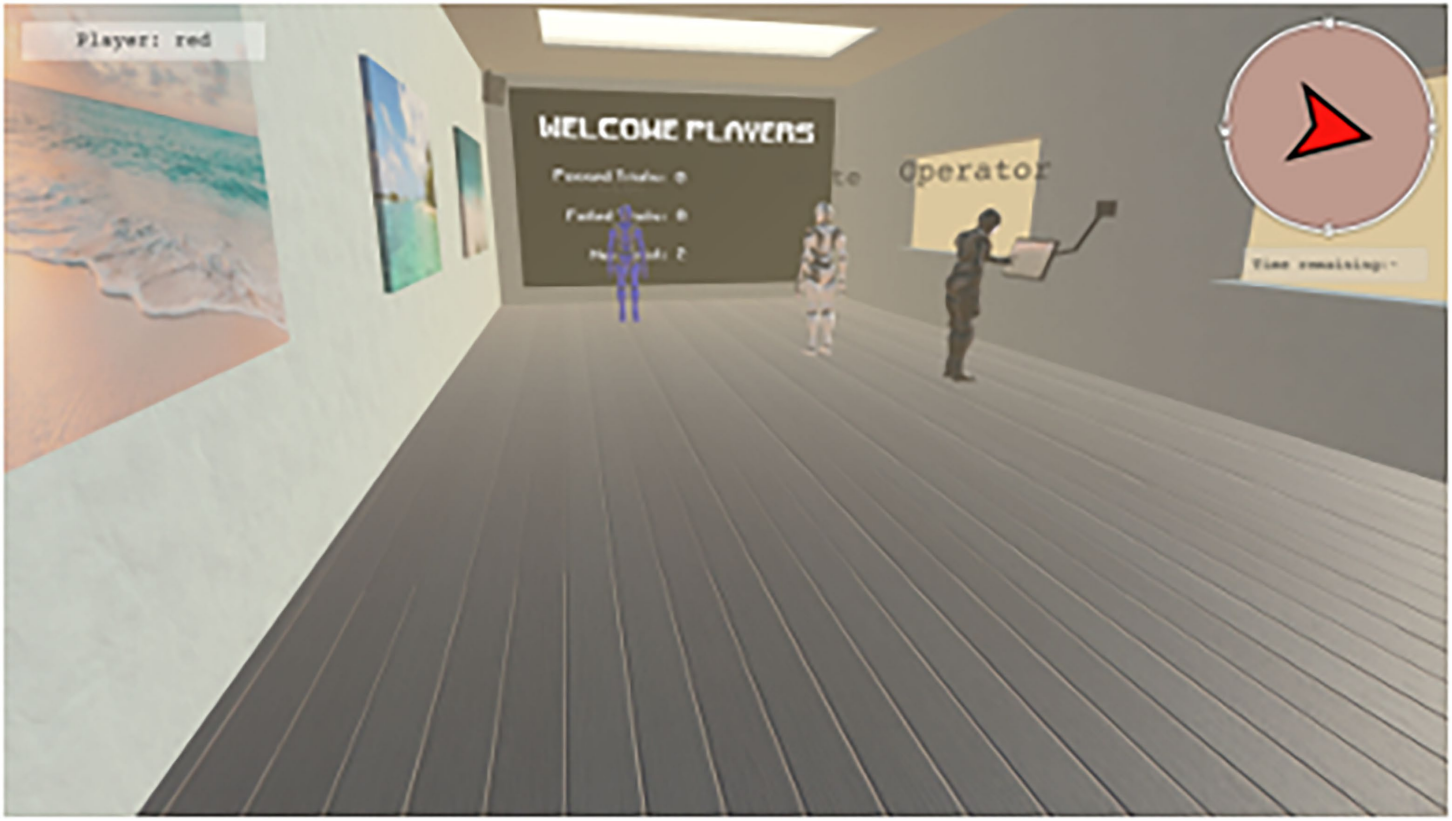
View of the game lobby. Participants were embodied as humanoid avatars, whose color was the same as the participant’s name in the experiment (the participant playing as the drone operator was referred to as “Operator”). Following each trial, participants were teleported to a game lobby where participants received feedback regarding the previous trial, summary of how many trials were successful/unsuccessful, and a timer to indicate when the next trial was to begin.

Ground players controlled humanoid game avatars (Figure 3). Participants could control their avatars using their mouse to control heading direction, and the “W,” “A,” “S,” and “D” keys on the keyboard to control forward, left strafe, right strafe, and backward locomotion, respectively. When moving, the avatars would move at a rate of 10 m·s^−1^. To allow for finer control, the avatar’s movements can be reduced to 5 m·s^−1^ by holding down the “Left Shift” key. Ground players were responsible for corralling and containing the TAs within the containment area. Participants could influence TAs by moving their avatars within the “threatened” radius of the TAs (10 m), causing them to flee in the opposite direction. To facilitate coordination between the ground players and the drone operator, ground players were also given a compass which provided information about their direction of heading (Figure 2).

**Figure 4:**
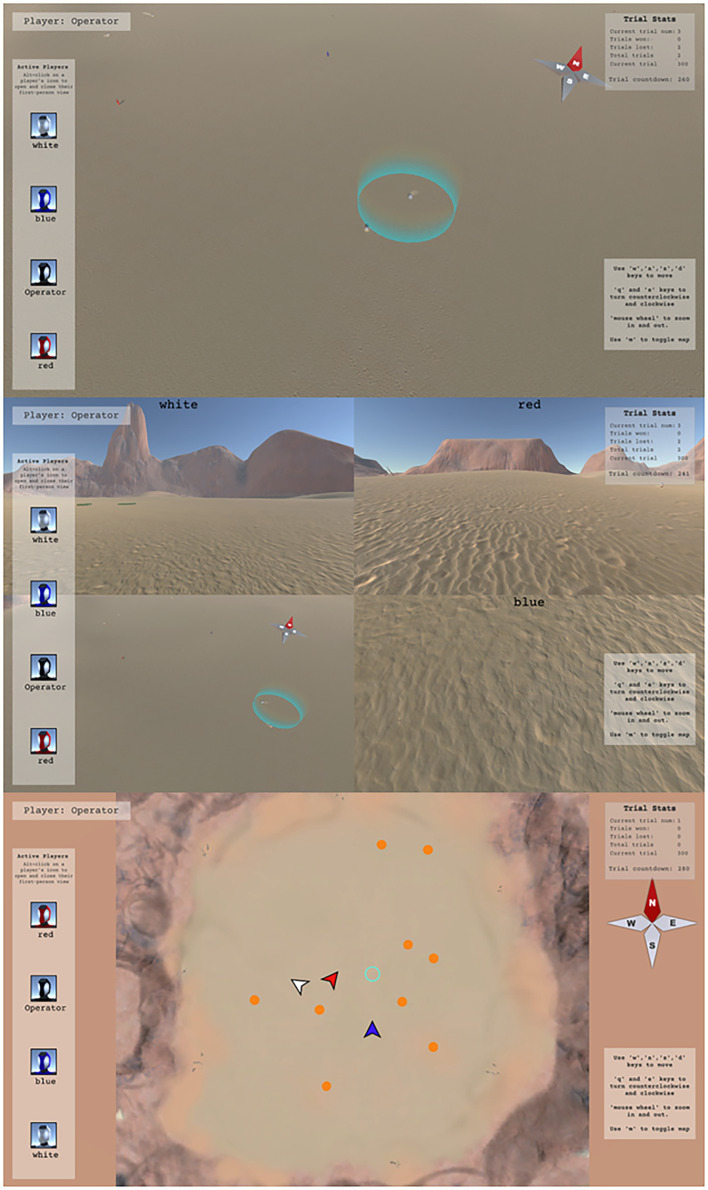
Drone operator game views. The participant playing as the drone operator had access to three different views of the task environment: fly-over view (top), ground player first-person view (middle), and map view (bottom). In fly-over view, participants used their mouse to control heading direction, and the “W,” “A,” “S,” and “D” keys on the keyboard to control forward, left strafe, right strafe, and backward camera movement, respectively. The drone operator could view the first-person perspective of up to three ground players (middle) by clicking on their player icon with the computer mouse while holding the “Left Alt” key. Finally, the map view (bottom) provided veridical information about the location of the ground players (the colored arrows) and TAs (the orange circles). The status of the TAs could also be viewed in map view (i.e., the color of the circle indicated if the TA was idle [orange], fleeing [red], contained [blue], and green [indicating all TAs were contained]).

The drone operator emulated a “spectator” common in many multiplayer FPS games. The participant playing as the operator had a user interface that enabled multiple different views of the task environment. Operators could “fly” around the environment in “fly-over” view (Figure 4, top), access the first-person view of any ground player (by selecting a ground player’s icon with the mouse while holding “Left Alt,” Figure 4, middle), or view a 2*D* global map of the task (by pressing the “M” key) (Figure 4, bottom). In fly-over view, participants used their mouse to control heading direction, and the “W,” “A,” “S,” and “D” keys on the keyboard to control forward, left strafe, right strafe, and backward movement, respectively, of their camera view. The drone operator could also increase/decrease the altitude of the camera using the mouse wheel.

The game was split into trials with a maximum duration of 5 min. If teams could keep all TAs contained within the containment area for 5 continuous seconds, the trial would end early, and participants would receive feedback that they have succeeded. Otherwise, if the trial time expired, the trial would end, and participants would receive feedback that they have failed. During each trial, participants had access to how much time was remaining. At the start of each session, and between each game trial, participants’ game avatars were placed into a game lobby which overlooked the game field (Figure 3). The game lobby contained a display screen which showed participants how many trials were completed, including the number of which resulted in success/failure. This screen also displayed whether the preceding trial led to success or failure. Between trials, participants remained in the game lobby for 10 s.

When a trial initiated, ground players were randomly placed within 100 m of the containment area, whereas TAs were randomly placed within 180 m of the containment area. When left unperturbed, the TAs exhibited Brownian motion dynamics, in that a random force between 0 to 60 N was applied to the TA in a random (*x*, *y*) planar direction at a rate of 1 Hz (TAs had 1 kg mass). When a participant’s avatar was within 10 m of a TA, an additional force was applied in the direction directly away from the participant’s avatar. This force was inversely proportional to the distance between the participant’s avatar and the TA (with a maximum force of 450 N). Both the Brownian and repulsive forces were applied until the TA reached a maximum velocity of 10 m·s^−1^.

The TAs also provided visual feedback regarding their status to participants by changing the color of the ring located around the middle of their body. The ring-light was orange when the targets were unperturbed (and hence only exhibited Brownian dynamics), red when fleeing a nearby participant avatar, blue when the TA was contained within the containment area, and green when all TAs were contained within the containment area.

#### Task manipulations

The task was manipulated in three ways. First, the number of targets that teams had to locate, corral, and contain was set to either 9 or 18 targets (*Target Number*; Figure 4, bottom for an example initial arrangement of 9 targets). Second, the visibility of the game environment was altered *via* the presence of game fog (*Visibility*). In the absence of fog, ground players had near perfect visibility and could see >150 m within the game space (Figure 2, top). In contrast, when fog was present, ground player visibility was restricted to being able to see clearly for approximately 10 m (Figure 2, bottom). Fog also hindered the operator’s ability to see the environment using fly-over view. Third, for some trials, a new TA would randomly appear towards the end of the trial (i.e., in the last 90 s, or after all TAs were contained, whichever occurred first; *Task Perturbation*).

Participants completed four sessions. For each session, teams completed two blocks, each consisting of 8 trials representing all possible combinations of the task manipulations (*Target Number*, *Visibility*, *Task Perturbation*). For the first two sessions, the drone operator was played by a different participant for each block (i.e., by the end of the second session, all participants would have had experience playing as the drone operator). Following the second session, a participant was randomly selected to play as the drone operator for the remaining two sessions. For one team, one participant did not have practice playing as the drone operator during the first two sessions (due to experimenter error). This person was designated as the operator for the third and fourth session for this team. For the first three sessions, trial order for each block was pseudo-randomized. For the fourth and final session, all teams completed the same random trial order.

### Procedure

Teams completed four, 90-min sessions. For this paper, only data from the fourth and final session are presented as we were predominately interested in skilled-level performance. Participants received $30 AUD for each session, and a bonus $40 AUD for completing all four sessions with the potential to receive an additional $50 AUD if their team had the best performance in Session 4 (assessed as the team who completed the most trials successfully and most rapidly in the case of a tie). Payment was provided in the form of an e-Gift Card.

Each participant was either assigned to be a ground player, or the drone operator. Each participant was also assigned a unique color to be used throughout the study which fellow participants could use to address a particular participant in the study (i.e., red, black, blue, or white). For each session, an e-mail was sent 30 min prior to the scheduled meeting time containing the link to the Zoom meeting as well as instructions for how to control their in-game avatars. Upon entering the Zoom meeting, participants were renamed to their assigned color by the experimenter and were told to keep their video cameras turned off. Once all participants reviewed the task instructions and turned their video camera off, the experimenter began audio recording. The experimenter’s microphone was muted during game play. For the experiment, an audio file for each participant, the experimenter, and the server hosting the experiment environment (for trial audio tones used to segment trials from the session long audio recording) was recorded as well as a combined audio stream of all participants in the team. Both the individual participants’ and teams’ combined audio files were recorded to facilitate audio transcription and verbal/conversation coding.

Following audio setup, participants were asked to launch the Unity application and connect to the game server. After it was confirmed that all participants had joined the game session and that their internet connection was stable, the 16 game trials commenced. Participants were told to complete the trials as fast as possible. In the event a player disconnected from the experiment (e.g., due to an unreliable internet connection), the experiment session was paused and the experimenter troubleshooted the issue. Upon the participant rejoining the experiment, the experimenter would restart the trial from the beginning. In the rare occasion a participant disconnected when the trial was about to be completed (e.g., the TAs were contained but not yet for the full 5 s), the experimenter would allow the trial to finish. This was done so not to frustrate the participants who were about to successfully complete a trial. Upon the participant rejoining the experiment, the experimenter would then proceed to the next trial.

After completing all trials or after the 90-min session time had elapsed, the experimental session was stopped, and the Zoom call ended. This procedure was followed for all four sessions. Following the fourth and final session, participants were debriefed about the purpose of the study and thanked for their participation. The recruitment procedures and study methodology were approved by the Macquarie University Human Research Ethics Committee.

### Audio data extraction and communication transcription

The audio stream for each session was segmented into 16 separate audio files, representing each trial, for each participant’s audio file. This was done in a semi-automated manner using the Aubio python package^3^ to automatically detect audible tones which were briefly played just before the start (900 Hz) and immediately following the end (1,100 Hz) of each trial.

For each individual audio file, a voice activity detection (VAD) library was used (WebCRTVAD)^4^ to determine which participant(s) spoke within 30 ms windows. The result was a binary time series (0 = silence, 1 = speaking) for each participant, for each trial. This procedure was performed using both “mid-high” and “mid-low” VAD parameter settings, resulting in the same pattern in the time series. For the analyses employed here, the time series was extracted using the mid-high VAD parameter settings.

The audio files were also transcribed in phases to ensure accuracy. First, the audio files were sent to an automated transcription service (Otter.ai, Los Altos, CA, United States).^5^ These automated transcripts were then edited by two independent transcribers to correct for any errors or omissions. Para-linguistic features were also transcribed such as laughter and verbal ticks (e.g., ‘umm’). Unless otherwise noted, all analyses included these features.

Following transcription, the transcripts were coded to identify (1) the speaker, (2) the recipient, and (3) whether the utterance was task-related or task-unrelated. The transcripts were coded using NVivo 12. The transcriptions had an inter-rater reliability of 0.90. The codes for speaker and recipient was 0.87, and task-related vs. unrelated was 0.92. For differences in the number of words/utterances attributed to a particular code, the average length was used.

### Measures

All completed trials (unsuccessful and successful) were included in analysis. The following measures were computed. Only data from the fourth session was analyzed.

#### Team performance

A team’s performance was assessed with two measures. First, performance was assessed by the trial’s duration in seconds (*Trial Duration*), where lower values indicated faster trial completion times with an upper ceiling value of 300 s for failed trials, representing the maximum trial duration. Second, *Containment Rate* was calculated as the number of TAs that were contained at the end of the trial, divided by the trial duration. Here, higher values corresponded to better team performance.

#### Team division of labor

A secondary team-level measure to assess team performance was to quantify the extent to which teams divided labor by partitioning where ground players searched. This division of labor was quantified as the proportion of the search area that was overlapping between two or more players. Here, 0% would indicate that teams cleanly partitioned the search space, while 100% would indicate that all participants searched the same locations.

**Figure 5:**
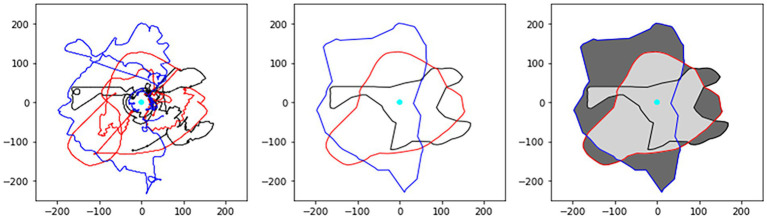
Illustration of how proportion overlap of player search was calculated. The position time series of each participant’s avatar (black, red, blue) (left) is submitted to the *alphashape* python toolbox. Concave polygons for each participant are then generated (middle), which represent the participants’ search areas. Polygon “fragments” are then obtained by taking the intersection of each pair-wise combination of participant search areas and then combined (light grey polygon in right panel). The proportion overlap of a team’s search area is then computed as the area of the light grey polygon divided by the total spread (represented as the sum of the dark grey and light grey polygons in the right panel). The cyan circle represents the location of the containment area. Adapted from Nalepka et al. (2022).

For each participant, a bounding polygon which encapsulated the participant’s avatar movements was created using the *alphashape* python toolbox^6^ to quantify each participant’s search area. The construction of the bounding polygon is an iterative process whereby a circle with radius *r*^−1^ is “rolled” along the extremities of the dataset, producing polygon edges whenever two data points intersect the circle. This process is repeated until the tightest-fitting single polygon is found (see Figure 5). Failure for *r*^−1^ to converge will result in the convex hull being used as the bounding polygon. The convex hull represents the smallest possible convex bounding polygon (whose shape is akin to a rubber band wrapped around pegs on a pegboard). Once the bounding polygons were created, the proportion of the search area that was overlapping was measured as the area spanning the overlapping polygons divided by the total search area. For this measure, each participant’s movement time series was downsampled to 5 Hz and the first second was deleted to remove any transient periods in behavior.

#### Magnitude of verbal communication

The magnitude of verbal communication was computed as the proportion of time a given participant was speaking during a trial – computed from the binary speaking time series. Separate values were computed for the drone operator and the ground players. For the three ground players, the proportions were averaged to create a singular value for the trial.

#### Structural dynamics of verbal communication

CatRQA (Dale and Spivey, 2006) was used to measure how the dynamics of team communication was impacted by the role participants adopted as well as the various task manipulations. CatRQA was employed at three different levels: measuring the communication dynamics of the drone operator, the ground players, and the dynamics at the team level (Figure 1). For the drone operator, catRQA was applied on the binary communication time series, described above, where “1” indicated that the operator was speaking, otherwise ‘0’ (sampled at 30 Hz). For the three ground players, a time series was constructed (sampled at 30 Hz), where if a ground player was speaking, the sample was coded with a unique identifier (i.e., “1,” “2,” “3”). If no ground player was speaking, the sample was coded as “0.” If two or more ground players were speaking, the sample was coded as “4.” At the team level, a similar procedure was followed, whereby speech by the drone operator was coded as “1,” “2–4” for the ground players, and “5” if two or more participants were speaking at the same time – other “0” for silence.

CatRQA was conducted for each time series for each trial. In short, catRQA identifies the dynamics of a system by discerning (1) whether the states of the system recur over time, and, if recurrent, (2) the degree to which the patterning of recurrences are highly regular or deterministic (Richardson et al., 2014). This analysis is done by performing statistics on recurrence plots (RPs) which, in the case of catRQA, is a 2*D* Boolean matrix with the considered time series presented on the *x*- and *y*- axes. To ensure that any recurrences are due to patterns in participant speaking behaviors, any periods of silences (i.e., data coded as “0”) was recoded with a random, non-repeating number prior to analysis.

Two catRQA measures were used to assess the dynamics of participant communication (the two catRQA measures most employed in the literature; (Coco and Dale, 2014; Richardson et al., 2014; Gorman et al., 2020). The first measure was the percentage of recurrent points (%REC) in the generated RPs. This measure captures the degree to which the same states of behavioral communication are repeated over time. The second measure was the percent of recurrent points that formed diagonal lines within the generated RPs, where such lines corresponded to sequences of recurrent points. This latter measure is referred to as percent determinism, or %DET, because it captures whether the same sequences of recurrent states are repeated over time. Here, high %DET corresponds to a more structured or deterministic pattern of behavioral communication.

## Results

All ten teams completed the experiment. For each dependent measure, 2 (*Target Number*: 9, 18) × 2 (*Visibility*: no-fog, fog) × 2 (*Task Perturbation*: No, Yes) repeated measures ANOVAs were utilized. Post-hoc analyses were corrected using the Bonferroni correction. Violations to sphericity were corrected using the Greenhouse–Geisser correction. For some dependent measures (e.g., trial duration), the assumption of normality was violated due to ceiling or floor effects. Given the robustness of ANOVAs to violations of normality, the data was not transformed prior to analysis.

### Team performance

**Figure 6:**
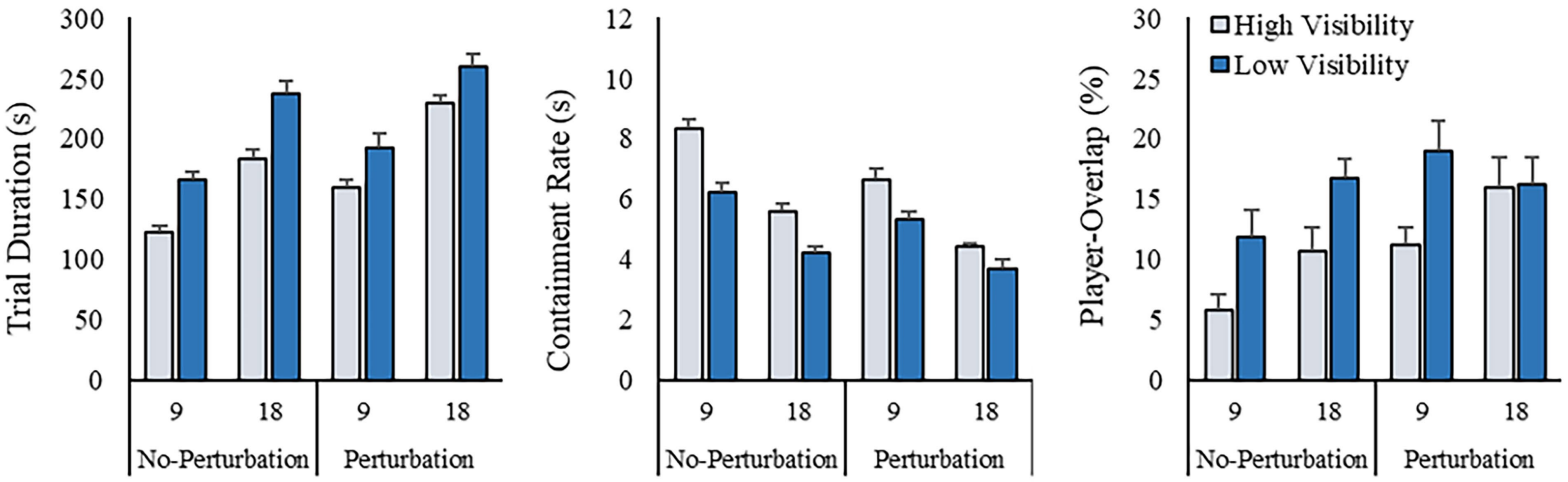
Summary of team performance and division of labor measures. Results are reported as a function of the different manipulations (target number, visibility, and task perturbation). Error bars represent the standard error of the mean.

A detailed summary of the means and standard deviations for each team performance measure as a function of condition are reported in Figure 6. As can been seen from this figure, teams completed trials faster when there were 9, as opposed to 18, TAs (*F* (1, 9) = 308.32, *p* < 0.001, *η_p_*^2^ = 0.97), when there was no environmental fog (*F* (1, 9) = 43.95, *p* < 0.001, *η*_p_^2^ = 0.83), and when no additional TA was added to the trial (*F* (1, 9) = 91.06, *p* < 0.001, *η*_p_^2^ = 0.91). There were no significant two-way (all *F* (1, 9) < 4.56, *p* > 0.061, *η*_p_^2^ < 0.34) or three-way interactions (*F* (1, 9) = 0.928, *p* = 0.36, *η*_p_^2^ = 0.09).

Similarly, the rate that participants contained the TAs was higher when there were 9, as opposed to 18, TAs (*F* (1, 9) = 197.70, *p* < 0.001, *η*_p_^2^ = 0.96), when there was no environmental fog (*F* (1, 9) = 60.08, *p* < 0.001, *η*_p_^2^ = 0.87), and when no additional TA was added to the trial (*F* (1, 9) = 95.06, *p* < 0.001, *η*_p_^2^ = 0.91). In addition to the main effects, the *Target Number* × *Visibility* (*F* (1, 9) = 7.40, *p* = 0.024, *η*_p_^2^ = 0.45), *Target Number* × *Task Perturbation* (*F* (1, 9) = 5.65, *p* = 0.041, *η*_p_^2^ = 0.39), and *Visibility* × *Task Perturbation* (*F* (1, 9) = 5.92, *p* = 0.038, *η*_p_^2^ = 0.40) interactions were also significant. As can be seen in Figure 6, participants contained TAs at a greater rate when there was 9, as opposed to 18 TAs, both when there was, and there was no fog present (all *p* < 0.01) – however, this difference was greater when there was clear visibility. Similarly, regardless of the *Task Perturbation* manipulation, participants contained the TAs at a greater rate when there was 9, as opposed to 18 TAs (all *p* < 0.01) – however, this difference was greater when there was no task perturbation introduced. Similarly, regardless of Task Perturbation, participants contained the TAs at a greater rate when there was no fog present than when there was fog present (all *p* < 0.02) – however, this difference was greater when there was no task perturbation introduced. There was no significant three-way interaction (*F* (1, 7) = 0.07, *p* = 0.804, *η*_p_^2^ = 0.01).

### Team division of labor

Teams were able to divide labor more efficiently (i.e., there was less overlap in where ground players moved) when there was no environmental fog (*F* (1, 9) = 33.52, *p* < 0.001, *η*_p_^2^ = 0.79), and when no task perturbation was introduced (*F* (1, 9) = 17.208, *p* = 0.002, *η*_p_^2^ = 0.66). There was no main effect for *Target Number* (*F* (1, 9) = 3.82, *p* = 0.082, *η*_p_^2^ = 0.30), nor were there any two-way (all *F* (1, 9) < 1.81, *p* > 0.211, *η*_p_^2^ < 0.17) or three-way (*F* (1, 9) = 3.41, *p* = 0.098, *η*_p_^2^ = 0.28) interactions.

### Magnitude of verbal communication

As expected, the number of utterances produced by participants playing as the drone operator increased when environmental fog was present (*F* (1, 9) = 42.67, *p* < 0.001, *η*_p_^2^ = 0.83). This difference was greater when 18 TAs had to be corralled and contained, compared to when 9 TAs were present, although the *Target Number* × *Visibility* interaction was not significant (*F* (1, 9) = 4.24, *p* = 0.07, *η*_p_^2^ = 0.32). No other main effects or interactions were significant (all *F* (1, 9) < 1.04, *p* > 0.33, *η_p_*^2^ < 0.11).

The ground players also spoke more when environmental fog was present (*F* (1, 9) = 9.25, *p* = 0.014, *η*_p_^2^ = 0.51), as well when the number of TAs to contain increased (*F* (1, 9) = 6.75, *p* = 0.029, *η*_p_^2^ = 0.43). In addition to the main effects, there were marginally significant *Target Number* × *Visibility* (*F* (1, 9) = 4.37, *p* = 0.066, *η*_p_^2^ = 0.33) and *Target Number* × *Task Perturbation* (*F* (1, 9) = 4.79, *p* = 0.056, *η*_p_^2^ = 0.35) interactions. The impact of *Visibility* and *Task Perturbation* was greater when 18 TAs, as opposed to 9, had to be contained, such that there was more speech when environmental fog was present, or if a TA was introduced in the trial. No other main effects or interactions were observed (all *F* (1, 9) < 0.19, *p* > 0.67, *η*_p_^2^ < 0.03). Table 1 for the means and standard deviations for each condition.

**Table 1:** Magnitude of verbal communication.

Measure	Fog		No fog	
	9 Bots	18 Bots	9 Bots	18 Bots
**Perturbation**nTT_OP_	0.59 (0.11)	0.63 (0.11)	0.49 (0.14)	0.22 (0.10)
nTT_GP_	0.20 (0.08)	0.19 (0.08)	0.22 (0.10)	0.24 (0.11)
**No perturbation**				
nTT_OP_	0.63 (0.11)	0.64 (0.11)	0.51 (0.15)	0.47 (0.16)
nTT_GP_	0.19 (0.08)	0.20 (0.08)	0.21 (0.10)	0.24 (0.11)

Values represent the mean for each condition (averaged across trial and team), with the standard deviations reported in parentheses.

### Structural dynamics of verbal communication

**Figure 7:**
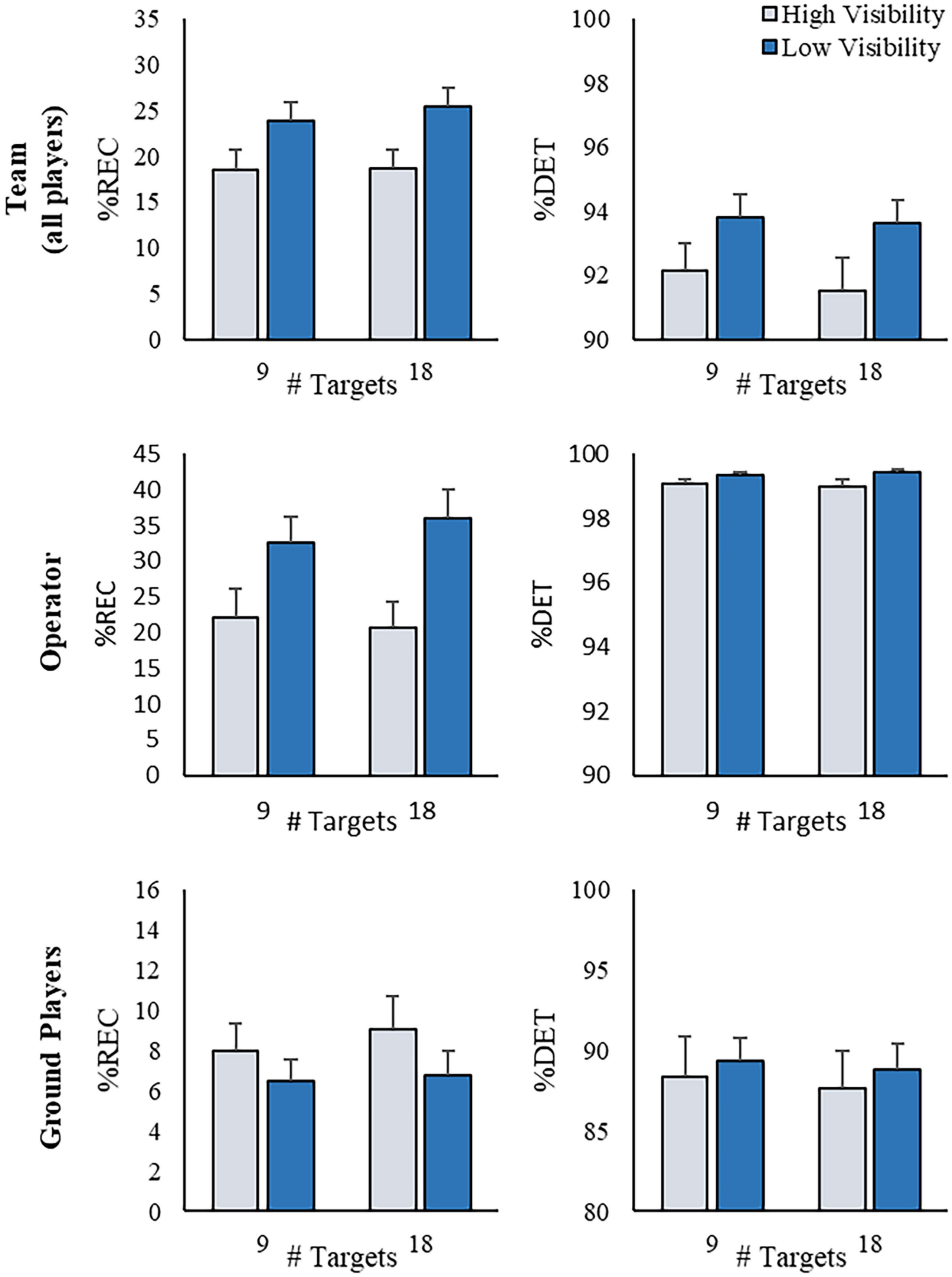
Summary of catRQA results. Mean %REC and %DET for the team (top), operator (middle) and ground player (bottom) communication data series as a function of the target number and visibility conditions. Error bars represent the standard error of the mean.

At the team level, recurrent quantification analyses revealed that %REC and %DET was impacted by the *Visibility* manipulation (%REC: *F* (1, 9) = 12.38, *p* < 0.01, *η*_p_^2^ = 0.579; %DET: *F* (1, 9) = 15.764, *p* < 0.01, *η*_p_^2^ = 0.638; Figure 7, top). The dynamical structure of the team’s communication was more recurrent and deterministic, meaning that teams communicated more and exhibited more routine patterns of communication, when there was environmental fog present. Further, team communication was more recurrent when teams corralled and contained 18, as opposed to 9, TAs (%REC: *F* (1, 9) = 5.942, *p* = 0.038, *η*_p_^2^ = 0.398). There was no main effect of *Task Perturbation* for %REC or %DET (both *F* (1, 9) < 2.03, *p* > 0.19, *η*_p_^2^ = 0.184), nor any interaction effects (all *F* < 1, *p* > 0.5).

When looking at the communication dynamics of the participant taking on the role of drone operator, similar results were observed (Figure 7, middle). Participants playing as the drone operator exhibited more recurrent dynamics (higher %REC: *F* (1, 9) = 55.85, *p* < 0.001, *η*_p_^2^ = 0.861) which was more deterministic (higher %DET: *F* (1, 9) = 12.638, *p* < 0.01, *η*_p_^2^ = 0.584) when environmental fog was present, than when visibility was clear. In other words, when environmental fog was present, the drone operator spoke more and within defined sequences. There was also a significant *Target Number* × *Visibility* interaction for %REC (*F* (1, 9) = 6.253, *p* = 0.034, *η*_p_^2^ = 0.410). Decomposing the interaction, the effect of *Visibility* was only significant when task visibility was low (i.e., fog was present; *p* < 0.001), and not when task visibility was high (*p* > 0.05).

A marginally significant *Target Number* × *Visibility* interaction was also found for %DET (*F* (1, 9) = 5.016, *p* = 0.052, *η*_p_^2^ = 0.358). There were no main effect of *Task Perturbation* for %REC or %DET (both *F* (1, 9) < 1, *p* > 0.5), nor any other interaction effects (all *F* (1, 9) < 2.475, *p* > 0.15, *η*_p_^2^ < 0.216).

Interestingly, the catRQA analysis for the ground player communication time series (see Figure 7, bottom) revealed a very different pattern of results. Specifically, ground player communication was significantly less recurrent (i.e., ground players communicated with each other less) when environmental fog was present (*F* (1, 9) = 9.369, *p* < 0.02, *η*_p_^2^ = 0.51) – the opposite of what was found for the operator and at the team-level. Further, there was no effect of *Target Number* or *Visibility* on %DET for ground players (both *F* (1, 9) < 3.507, *p* > 0.094, *η*_p_^2^ = 0.280). Finally, although there was no main effect of *Task Perturbation* for either %REC or %DET (all *F* < 2.003, *p* > 0.191, *η*_p_^2^ < 0.182), there was a significant *Target Number* × *Task Perturbation* interaction for %REC (*F* (1, 9) = 5.458, *p* < 0.05, *η*_p_^2^ = 0.378). Similar to what was found for the operator and at the team level, the structure of ground players’ communication was significantly less recurrent (i.e., lower %REC) when the task required the containment of 9, as opposed to 18, TAs (*F* (1, 9) = 10.251, *p* < 0.02, *η*_p_^2^ = 0.532). There were no other interactions for either %REC or %DET (all *F* < 2.003, *p* > 0.191, *η*_p_^2^ < 0.182) for the ground players communication time series.

### Relationship between team performance and communication

Finally, linear regression was employed to determine whether there was any additional association between the magnitude, symmetry, and dynamic structure of verbal communication and overall team performance and coordination. Specifically, linear regression was employed to evaluate whether any of the above detailed communication measures predicted team performance (i.e., *trial duration* and *containment rate*), after accounting for the variance associated with *Target Number*, *Visibility*, and *Task Perturbation*. The regression took the following form,


$$Y_{i}=\beta_{0}+f\left(X_{i},\beta_{X}\right)+z\beta_{z}+u_{i}+\varepsilon_{i}$$

where the dependent variable, $y_{i}$ was either *trial duration*, *containment rate*, or *team division of labor*, $\beta_{0}$ was the model intercept, *f* (*X_i_, β_X_*) represents the independent variables and associated coefficients for *Target Number*, *Visibility*, *Task Perturbation*, and corresponding two-way and three-way interaction factors, $u_{i}$ was the random effect (intercept) for each team, $\varepsilon_{i}$ was the additive error term (i.e., remaining variance unaccounted for), and $z\beta_{z}$ was the verbal communication measure and coefficient of interest (e.g., communication magnitude, %REC, etc.).

For the magnitude of ground player communication, the analysis revealed a significant positive relationship for *trial duration* (*β* = 149.89, *σ*_m_ = 63.85, *z* = 2.35, *p* < 0.02, *CI* = [24.76, 275.03], *f^2^* = 0.113) and *containment rate* (*β* = −005, *σ*_m_ = 0.002, *z* = − 2.42, *p* < 0.02, *CI* = [−0.009, −0.0009], *f^2^* = 0.104), with a greater magnitude of ground player communication resulting in worse performance. No such relationship existed regarding communication by the drone operator (both *trial duration* and *containment rate p* > 0.495). No other significant relationships were observed for the magnitude of task-relevant communication.

Consistent with the relationship between the task performance measures and the magnitude of ground player communication, both *trial duration* and *containment rate* were also positively related with the %REC of ground player communication (*β* = −3.34, *σ*_m_ = 1.31, *z* = −2.55, *p* < 0.02, *CI* = [−5.90, −0.77], *f^2^* = 0.069 and *β* = −0.071, *σ*_m_ = 0.022, *z* = −3.2, *p* < 0.01, *CI* = [−0.114, −0.027], *f^2^* = 0.088, respectively), with more recurrent ground player communication associated with worse task performance. There was, however, no significant relationships between %DET of ground player communication and trial duration or containment rate (both *p* > 0.51).

Interestingly, when considering the communication dynamics at the team-level, the relationships were reversed. Specifically, there was a significant negative relationship between *containment rate* and %REC of the team-level communication (*β* = −0.034, *σ*_m_ = 0.017, *z* = −2.01, *p* < 0.05, *CI* = [−067, −0.0001], *f^2^* = 0.062), such that the more recurrent a team’s communication dynamics, the faster they were at corralling and containing the TAs. There were no significant effects for any of the other team-level or drone operator catRQA communication measures (all *p* > 0.14).

## Discussion

The current study explored team performance and communication dynamics in a custom-built cooperative first-person multiplayer video game. The *Desert Herding* game required teams of four participants to work together to locate, corral, and contain evasive TAs scattered throughout a large desert environment. Participants playing the game took on one of two distinct roles – three ground players who could navigate and interact with the game environment, and one drone operator whose role was like a “spectator.” Although the operator could not directly interact with the environment, they had access to veridical information about the task. These role differences created a natural knowledge asymmetry between the players, which became exaggerated when teams were exposed to environmental fog, which uniquely impacted ground players’ ability to locate and corral the TAs but had no impact on the drone operator’s ability to communicate the states of task-relevant objects using the map.

As expected, the inclusion of environmental fog in the game challenged teams’ ability to complete the Desert Herding game. Specifically, the time needed for ground players to locate, corral, and contain the TAs increased when visibility was poor. Additionally, the movements of players when searching was not as efficient when exposed to fog, as evidenced by the finding that the ground players’ movement areas overlapped more when searching for TAs. To compensate for the challenges to task performance that the fog caused, both ground players and the drone operator increased the number of verbal statements that were produced to build and maintain awareness of the locations of the TAs, and of other players. Further, due to the knowledge asymmetry that existed between the ground players and the drone operator, the presence of environmental fog reorganized the communication dynamics between the participants. Specifically, in the more difficult task condition, ground players directed more of their conversation to the drone operator (see Supplemental Information for additional analyses). Variation in the number of TAs teams had to corral (9 or 18; or when a new TA was included towards the end of the trial) did not have an impact on the structure of how teams communicated. This is most likely because such manipulations did not challenge a team’s ability to maintain TSA.

The reorganization of the conversation dynamics of teams due to changes in task visibility was supported by the catRQA results. At the team level, greater coordination between team members was observed when fog was present (as assessed by both %REC and %DET). However, when the conversation dynamics of teams was decomposed into their respective roles, the drone operator was responsible for the increase in coordination because their statements were more frequent (as assessed by %REC) and more deterministic (as assessed by %DET). In contrast, the communication dynamics within the ground players was less structured when fog was present. Indeed, the results from the linear regression analysis showed that, after accounting for the task manipulation effects, greater and more recurrent verbal communication amongst ground players was associated with worse task performance.

The inclusion of fog necessitated an increased dependence of language to communicate the locations of TAs. The catRQA results indicate that the communication patterns teams adopted became more stable because the operator played a larger role in maintaining TSA. However, this coordination strategy did not mitigate the impacts of environmental fog on team performance. This may be due to the difficulty of language to communicate nuanced relationships between the ground players, the TAs, and their positions in reference to the containment location (e.g., communicating the shape of a TA cluster and how best to corral them). Anecdotally, the participant playing as the drone operator often dictated what actions ground players should take but also appeared to communicate less about how ground players were positioned in relation to each other. This may be a contributing factor why participants were not as efficient searching for TAs when fog was present. Further, the reliance on the drone operator introduced a coordination bottleneck as ground players were not able to meaningfully contribute to building TSA due to the lack of visibility. This bottleneck required the operator to make serial decisions about which individual ground player a verbal statement should be directed towards. In contrast, when no fog was present, ground players had a greater ability to perform tasks independently without the guidance of the operator, which then enabled a team’s search to be more like a parallel process. In recent work (Nalepka et al., 2022), the challenges imposed by fog could be mitigated by giving direct access of the in-game map to the ground players – in this case, through a *head-up display* (*HUD*). This work demonstrated that when ground players are given a HUD, there was no impact of task visibility on team performance.

This study, consistent with previous research, documents the dynamics which define effective team performance and coordination. However, in the area of *human-autonomy teaming* (*HAT*; O’Neill et al., 2020), there is less research focusing on how such dynamic patterns can be generated by artificial agents that could serve as synthetic teammates (Nalepka et al., 2021a). Applications for HAT include providing artificial agents that exhibit human-like behaviors to facilitate team training exercises (Rigoli et al., 2020), or adaptable agents that can enhance overall team functioning. Previous research investigating HAT in a three-person air reconnaissance task has documented that participants conversing with an artificial agent, *via* a text-based system, exhibited patterns of communication that were more rigid compared to all-human teams (Demir et al., 2019). This rigidity was shown to result in worse performance when teams had to overcome unexpected barriers during the task (Demir et al., 2019). More recent work developing artificial agents using deep reinforcement learning (i.e., agents whose state-action policies are represented using a multi-layer neural network training to optimize a reward function) has shown the potential to developing agents that can work alongside humans in cooperative, movement-based tasks (Carroll et al., 2019; Nalepka et al., 2021a).

Of particular interest is training artificial agents capable of spoken conversation. In this way, participants engaged in the task can use natural spoken language to interact with an artificial agent (and receive verbal feedback), as opposed to relying on text-based communication like in previous research (Demir et al., 2019). Using the task explored here, conversational agents could assume the role of the drone operator and provide guidance to ground players or engage in the task directly as a ground player. Further, teams could be composed of multiple artificial agents who can not only converse with humans, but with each other. Previous research has explored the development of artificial agents that can comprehend (e.g., (Blukis et al., 2018; Misra et al., 2018)) and produce language input (e.g., (Abramson et al., 2020, 2021)) in instruction-following tasks using supervised learning techniques. However, this research, as well as most commercially available conversational platforms used today (e.g., Amazon’s Alexa, Google’s Dialogflow) rely on dyadic, question-response interactions (Seering et al., 2019). To enable conversational agents to function in team-based tasks such as the one employed in this study, such agents need to be able to monitor the conversations of all team members and to develop expertise in anticipating what and when information will be requested by the team members (Simpson and Crone, 2021).

For artificial agents to become effective teammates within mixed human-machine teams, future research needs to simultaneously explore the coordination dynamics that differentiate high- from low-performing teams in various task conditions, as well as develop and assess algorithms which enable artificial agents to act and converse with their human teammates. Given the challenges associated with remote collaboration, teamwork within online, multiplayer video games are a promising paradigm to explore human- and human-autonomy teaming. Although derided by the FPS gaming community due to their incompetence, artificial agents (or “bots”) of the future have the potential to contribute meaningfully as synthetic teammates (or opponents) to enhance team interactions.

## Data availability statement

The raw data supporting the conclusions of this article will be made available by the authors, without undue reservation.

## Ethics statement

The studies involving human participants were reviewed and approved by Macquarie University Human Research Ethics Committee. The patients/participants provided their written informed consent to participate in this study.

## Author contributions

All authors contributed to the study’s conceptualization and methodology. JS contributed to investigation and data curation. JS, PN, and MR contributed to visualization and writing the original draft. All authors contributed to manuscript review and editing. DR and MR contributed to supervision. PN, RK, MD, ER, and MR contributed to funding acquisition. All authors contributed to the article and approved the submitted version.

## Funding

This work was supported by the Australian Department of Defence, Defence Science and Technology Group (partnership grant MyIP8655) and Human Performance Research Network (HPRNet, partnership grant ID9024). PN was supported by the Macquarie University Research Fellowship and MR was supported by the Australian Research Council Future Fellowship (FT180100447).

## Conflict of interest

The authors declare that the research was conducted in the absence of any commercial or financial relationships that could be construed as a potential conflict of interest.

## Publisher’s note

All claims expressed in this article are solely those of the authors and do not necessarily represent those of their affiliated organizations, or those of the publisher, the editors and the reviewers. Any product that may be evaluated in this article, or claim that may be made by its manufacturer, is not guaranteed or endorsed by the publisher.

## Footnotes

^1^ https://www.speedtest.net

^2^ https://github.com/vis2k/Mirror/

^3^ https://pypi.org/project/aubio/

^4^ https://github.com/wiseman/py-webrtcvad

^5^ https://otter.ai

^6^ https://pypi.org/project/alphashape/
