# Supplementary material for: Conversation dynamics in a multiplayer video game with knowledge asymmetry
Source: Front Psychol. 2022 Nov 3;13:1039431. doi: 10.3389/fpsyg.2022.1039431 (PMC9669907; doi:10.3389/fpsyg.2022.1039431)
Supplement: Supplementary file 1 [file Table_1.DOCX]

# SUPPLEMENTAL INFORMATION

**ADDITIONAL COMMUNICATION MEASURES**

### Magnitude of On-Task Communication

The magnitude of on-task communication was computed as the proportion of a given participant’s transcript that was coded as task-related utterances. Like the magnitude of verbal communication measure presented in the main manuscript, this measure was computed for the drone operator and the ground players (where, again, the average proportion was used for the ground players).

As expected, participants playing as the drone operator made more task-relevant statements when environmental fog was present, although this difference was marginally significant (*F*(1,9) = 5.03, *p* = .052, *η_p_*^2^ = .36). There was no main effect for *Target Number* (*F*(1,9) = 2.29, *p* = .165, *η_p_*^2^ =.20) and *Task Perturbation* (*F*(1,9) = 1.08, *p* = .327, *η_p_*^2^ = .11), as well as no significant interactions (all *F*(1,9) < 2.30, *p* > .163, *η_p_*^2^ < .21).

For participants completing the task as ground players, they made more task-relevant statements when environmental fog was present (*F*(1,9) = 6.21, *p* = .034, *η_p_*^2^ = .41). There was also a significant *Target Number* × *Visibility* interaction (*F*(1,9) = 9.22, *p* = .014, *η_p_*^2^ = .51). The effect of *Visibility* was significant if there were 18 TAs to contain (*t*(9)=3.49, *p* =0.028), but not when there was 9 TAs (all *t*(9)<1.48, *p* > .05). No other main effects nor interaction effects were significant (*F*(1,9) < 1.37, *p* > .271, *η_p_*^2^ < .14).

### Symmetry of Verbal Communication

In addition to *categorical recurrence quantification analysis* (*catRQA*), two conversation symmetry measures were employed to quantify the balance (i.e., symmetry) of recipients for a verbal utterance. For the drone operator, *equality in talking partner* (Traeger et al., 2020) was used to determine how evenly distributed the operator’s utterances were to each of the ground players (acronymized as *ETP_OP_*). ETP_OP_ was calculated as

| ${ETP}_{OP}=\sum\frac{\vert\tau\left( OP,GPi \right)- \tau(OP,GPj)\vert}{\tau\left( OP,GPi \right)+ \tau(OP,GPj)}$ | (1) |
| --- | --- |

where $i\neq j=1, 2, 3$ and $|\tau\left( OP,GPi \right)- \tau(OP,GPj)|$, represents the difference (expressed as a positive value) in the amount of speech the *operator* (*OP*) directed towards each pairwise combination of *ground player* (*GP*) *i* and *j*, with the divisor, $\tau\left( OP,GPi \right)+ \tau(OP,GPj)$ , being the total sum of utterances towards ground players *i* and *j*. Hence, the $\frac{|\tau\left( OP,GPi \right)- \tau(OP,GPj)|}{\tau\left( OP,GPi \right)+ \tau(OP,GPj)}$, reveals whether the proportion of operator utterances were equally directed towards ground player *i* and *j*. After each pairwise combination was assessed, the average was taken. A value closer 0 would indicate that the operator spoke equally to each ground player, whereas a value closer to 1 would indicate that the operators’ utterances were asymmetrically distributed between the ground players.

For the ground players, the symmetry of communication was assessed by calculating the proportion of ground player utterances that were directed towards the drone operator (acronymized as $GP\to OP$). $GP\to OP$ was defined as

| $GP\to OP= \frac{\tau\left( g_{1},o \right)+\tau\left( g_{2},o \right)+ \tau(g_{3},o)}{\tau_{g_{1}+}\tau_{g_{2}+}\tau_{g_{3}}}$ | (2) |
| --- | --- |

where $\tau\left( g_{i},o \right)$ represents the total number of utterances by a ground player (*i* = 1, 2, or 3) directed towards the operator and $\tau_{g_{i}}$ represents the total number of utterances by all ground players.

For both symmetry measures (i.e., ETP_OP_, $GP\to OP$), utterances that were directed to everyone on the team was coded as being directed towards each participant individually. For instance, if a segment of communication in the transcript was coded as ‘RED_TO_ALL’, this would be coded as ‘RED_TO_WHITE’, ‘RED_TO_BLUE’, and ‘RED_TO_BLACK’ for purposes of the communication symmetry analysis. Any self-directed utterances were omitted from the computation of these measures.

Regardless of condition (all *F*(1,9) ≤ 4.38, *p* ≥ .066, *η*_p_^2^ ≤ .33), participants who played as the drone operator spoke to all ground players equally. In contrast, ground players directed their speech towards the drone operator more when environmental fog was present (*F*(1,9) = 50.24, *p* < .001, *η_p_*^2^ = .85). There was also a marginally significant *Target Number* × *Visibility* interaction (*F*(1,9) = 4.84, *p* = .055, *η_p_*^2^ = .35). The effect of the *Visibility* manipulation was greater when 18 TAs had to be corralled and contained then when there were 9 TAs. In other words, as task difficulty increased (via increased difficulty for ground players to perceive their surrounding environment), there was less inter-ground player communication. No other main effects or interactions were found (all *F*(1,9) < 3.96, *p* > .077, *η_p_*^2^ < .31).

| \| **Supplemental Table 1.** Magnitude and Symmetry Communication Measures \| \| \| \| \| \| \| --- \| --- \| --- \| --- \| --- \| --- \| \| Measure \| Perturbation \| \| \| \| \| \| Fog \| \|  \| No Fog \| \| \| 9 Bots \| 18 Bots \|  \| 9 Bots \| 18 Bots \| \| OnTask_OP_ \| 0.99 (0.02) \| 1.00 (0.00) \|  \| 0.95 (0.09) \| 0.97 (0.03) \| \| OnTask_GP_ \| 0.90 (0.17) \| 0.94 (0.08) \|  \| 0.87 (0.20) \| 0.80 (0.22) \| \| ETP_OP_ \| 0.22 (0.07) \| 0.15 (0.05) \|  \| 0.17 (0.08) \| 0.21 (0.09) \| \| GP→OP \| 0.74 (0.21) \| 0.79 (0.15) \|  \| 0.57 (0.26) \| 0.54 (0.15) \| \|  \| \| \| \| \| \| \|  \| No Perturbation \| \| \| \| \| \|  \| Fog \| \|  \| No Fog \| \| \|  \| 9 Bots \| 18 Bots \|  \| 9 Bots \| 18 Bots \| \| Measure \|  \|  \|  \|  \|  \| \| OnTask_OP_ \| 0.98 (0.04) \| 0.98 (0.04) \|  \| 0.93 (0.11) \| 0.96 (0.08) \| \| OnTask_GP_ \| 0.90 (0.11) \| 0.89 (0.22) \|  \| 0.85 (0.20) \| 0.83 (0.30) \| \| ETP_OP_ \| 0.18 (0.05) \| 0.18 (0.05) \|  \| 0.24 (0.13) \| 0.19 (0.10) \| \| GP→OP \| 0.69 (0.13) \| 0.76 (0.17) \|  \| 0.68 (0.22) \| 0.62 (0.26) \| \| *Note:* Values represent the mean for each condition (averaged across trial and team), with the standard deviation reported in parentheses. \| \| \| \| \| \| |
| --- | --- | --- | --- | --- | --- | --- | --- | --- | --- | --- | --- | --- | --- | --- | --- | --- | --- | --- | --- | --- | --- | --- | --- | --- | --- | --- | --- | --- | --- | --- | --- | --- | --- | --- | --- | --- | --- | --- | --- | --- | --- | --- | --- | --- | --- | --- | --- | --- | --- | --- | --- | --- | --- | --- | --- | --- | --- | --- | --- | --- | --- | --- | --- | --- | --- | --- | --- | --- | --- | --- | --- | --- | --- | --- | --- | --- | --- | --- | --- | --- | --- | --- | --- | --- | --- | --- | --- | --- | --- | --- | --- | --- | --- | --- | --- | --- | --- | --- | --- | --- | --- | --- | --- | --- | --- | --- |

# REFERENCES

Traeger, M. L., Sebo, S. S., Jung, M., Scassellati, B., & Christakis, N. A. (2020). Vulnerable robots positively shape human conversational dynamics in a human-robot team. *Proceedings of the National Academy of Sciences of the United States of America*, *117*(12), 6370–6375. https://doi.org/10.1073/pnas.1910402117
